# Addressing Chronic Malnutrition through Multi-Sectoral, Sustainable Approaches: A Review of the Causes and Consequences

**DOI:** 10.3389/fnut.2014.00013

**Published:** 2014-08-15

**Authors:** Kristina Reinhardt, Jessica Fanzo

**Affiliations:** ^1^School of International and Public Affairs, Columbia University, New York, NY, USA; ^2^Institute of Human Nutrition, Columbia University, New York, NY, USA; ^3^Center for Globalization and Sustainable Development, Earth Institute, Columbia University, New York, NY, USA

**Keywords:** stunting, chronic malnutrition, growth failure, multi-sector collaboration, nutrition-sensitive

## Abstract

Chronic malnutrition, including stunting, is an important example of a global challenge that spans multiple sectors, specifically health, agriculture, and the environment. The objective of this paper is to review current knowledge on the causes and consequences of chronic malnutrition and their relationship with multiple sectors. Understanding the causes includes approaching chronic malnutrition from the basic, underlying, and immediate levels. The causes reach from macro-level environmental influences to specific micronutrient intake. In order to effectively address stunting, it is important to understand the timing of stunting and the ability of individuals to catch up in terms of linear growth, cognitive ability, and immune function. The consequences of chronic malnutrition are transgenerational and they have an impact at the individual, community, and national level in the short- and long-term. There are still many gaps in knowledge regarding both the causes and consequences of chronic malnutrition, particularly when it comes to the interaction with agriculture and the environment, and understanding these gaps is important to addressing the burden of chronic malnutrition through evidence-based interventions.

## Introduction

The primary objective of this paper is to assemble current knowledge regarding chronic malnutrition in relation to multiple sectors. Sustainability must be at the forefront for each sector to address the challenges of chronic malnutrition. The paper will focus mainly on stunting as the most common manifestation of chronic malnutrition. To understand chronic malnutrition, we must define the syndrome, causes, and short- and long-term consequences. We will then discuss how these consequences can be addressed through evidence-based nutrition-specific and -sensitive interventions and governance. Furthermore, the contribution to the manifestation of chronic malnutrition by multiple sectors will be discussed.

As more global attention is directed toward combating stunting, there needs to be a clear understanding of what the nutrition community and the wider development community knows about stunting, how stunting is related to chronic malnutrition, and why stunting is used as an indicator for chronic malnutrition. This understanding is key for guiding future programing and policy. There are still many unknowns related to the causes and effects of chronic malnutrition that impact the way in which interventions are designed and implemented. Some of these unknowns are found within the biomedical realm. For example, we do not yet know how specific micronutrients contribute to growth failure and the ability and timing of children to catch up. Other unknowns include, which interventions are most effective during specific times in the lifecycle, particularly interventions from the agriculture and environment sectors.

Chronic malnutrition is defined as a form of growth failure that causes both physical and cognitive delays in growth and development. Stunting, also known as linear growth failure, is defined as the inability to attain potential height for a particular age, and it is the most common measurement used to identify chronic malnutrition. However, stunted growth is only one manifestation of chronic malnutrition. Compared to children who have been given optimal opportunities to grow and develop, a chronically malnourished child will be challenged to attain the same height, will likely not develop the same cognitive ability, and will have higher risk of poor health outcomes throughout life. The “window of opportunity,” or critical period of growth and development between pre-conception and 2 years of age, has been an important focus for the nutrition community (1). The window of opportunity is the period when a child is most sensitive to the impacts of poor nutrition.

Types of malnutrition fall into two general categories, acute and chronic. Acute malnutrition, most often demonstrated by wasting, is frequently seen in temporary or cyclical settings like emergencies, seasonal depressions, and highly infectious-disease environments. Globally, more children under 5 years of age are stunted than wasted; 165 million compared to 52 million in 2011, respectively (2). Yet, more children are likely to die from being wasted than stunted (3). There are specific, evidence-based protocols for the treatment of moderate and severe acute malnutrition (4). However, there are no standardized procedures for the treatment of chronic malnutrition or stunting. The causes of chronic malnutrition are multidimensional, as are the consequences, which can be both short-term and long-term.

The majority of stunted children live in low- and middle-income countries, with the highest proportion in African countries, where 35.6% of all children under 5 years are stunted, and the greatest absolute number in Asian countries, where 95.8 million children under 5 years are stunted (5). Globally, over the last few decades, the prevalence of stunting has decreased. However, prevalence of stunting in sub-Saharan Africa has remained stagnant compared to other regions. The global decline has mostly been driven by East Asia and the Pacific, where stunting declined by 71% between 1990 and 2011 (2). Currently, 80% of the total number of stunted children lives in just 14 countries; including India, Nigeria, and Pakistan (2). These countries have varied and contextual development challenges, which can broadly be rooted in high poverty, conflict (past and current), and/or natural disasters. These challenges have an impact on the likelihood of poor nutrition outcomes.

The causes of chronic undernutrition are multidimensional, which create many challenges in understanding the condition and finding solutions through interventions and policies. There is no single root cause of chronic malnutrition. The future strategies to address chronic malnutrition need the collaboration of multiple sectors and a variety of stakeholders in governments, non-government organizations, the donor community, and the private sector. This paper aims to capture and explain the multiple dimensions of chronic undernutrition and the importance of a multi-sectoral, sustainable response to improving nutrition that incorporates perspectives from agriculture, health, environment, water and sanitation, infrastructure, gender, and education.

## Definition

Stunting is the symptom most often used as a proxy for chronic undernutrition and is measured as a height- or length-for-age. Besides height deficits, other symptoms of chronic malnutrition include cognitive disability, decreased motor skills, and compromised immune function. The symptoms of chronic undernutrition can begin during conception and can have a lasting effect throughout the lifecycle. As a reaction to undernutrition *in utero*, a child may be born smaller and with an impaired immune system. Similarly, a child may respond to undernutrition in infancy and early childhood with a slower growth rate (6). These reductions can be considered a survival technique of the human body to adapt to harsh conditions.

Starting from conception, a child’s genetic potential interacts with environmental influences to affect health and nutrition outcomes. Environmental influences include the nutrients acquired *in utero*, from breastfeeding, and through the introduction of diverse complementary foods during weaning. Environmental influences also include negative effects, such as potential exposure to viral, bacterial, and parasitic infections due to poor sanitation and hygienic conditions. Due to the window of opportunity, the greatest growth velocity potential happens between conception and 2 years. This includes physical growth and the development of motor skills after birth. After 2 years, growth slows until puberty. The development of the musculoskeletal system requires specific nutrients to develop normally. From birth to 3 years, the brain grows in complexity and is highly sensitive to environmental influences. The neurological development process needs adequate availability of energy, protein, and fatty acids, as well as micronutrients including iron, iodine, zinc, and thiamin (7).

From a broader perspective, chronic malnutrition caused by the lack of specific nutrients at specific times in early childhood is most commonly associated with poor socioeconomic status, particularly in developing countries. The presence of stunting in an individual or community is not simply addressed by providing the correct amount of nutrients. The causes of chronic undernutrition become more complex when considering how and when these nutrients are delivered. As depicted in the UNICEF conceptual framework of malnutrition (8), consideration must be given to maternal nutrition, infant and young child feeding (IYCF) practices, food insecurity, access to health care, and the disease environment. It is important to note that stunting is a cumulative process, meaning that the percentage of children who are stunted increases up to the age of 2 years and then levels off (9).

In order to identify a child as stunted, the height of a child at a certain age is compared to a reference population measured at that age. The currently accepted reference population standards are based on the 2006 WHO Multicentre Growth Reference Study, which measured a cohort of children who were raised in favorable conditions for growth and development. The study was conducted over 6 years, following children from birth to 6 years of age. Children enrolled were sampled from Brazil, Ghana, India, Norway, Oman, and the USA. A critical finding was that the height of children by age is similar across the six sites. There was only about 3% variability in length between sites, which means, given the aforementioned optimal environment, children from different genetic and cultural backgrounds grow according to a similar trajectory until the age of 5 years (10). This finding is not entirely new; an earlier study on differences in height between children of different ethnic backgrounds showed minimal variation compared to the variation of environmental factors (i.e., the care environment or socioeconomic status) (11).

## Causes

**Figure 1:**
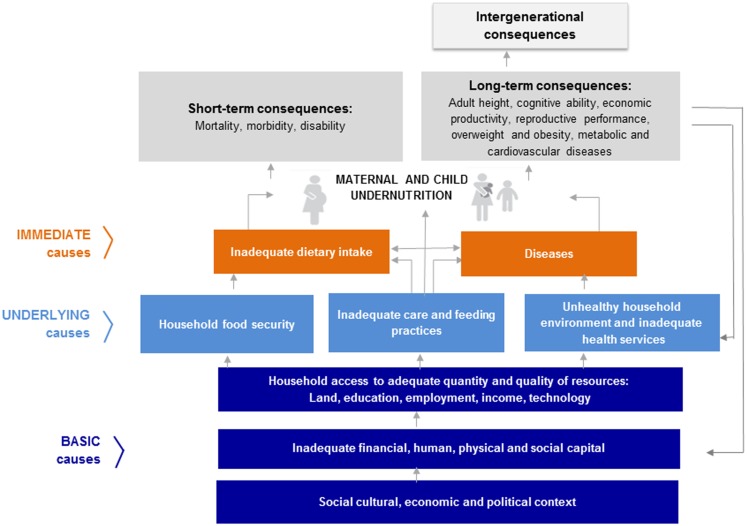
**The UNICEF conceptual framework of undernutrition is shown**. Source: UNICEF. *Improving Child Nutrition: The achievable imperative for global progress*. United Nations Children’s Fund; 2013. p. 4.

The UNICEF conceptual framework on undernutrition (Figure 1) is used to guide interventions from a multi-sectoral and multidimensional perspective, moving from macro to micro-levels of focus. The framework includes the basic, underlying, and immediate causes of malnutrition. The basic causes address systemic-level challenges reflecting the structural and political processes in each society, which includes social, economic, environmental, and political issues that lead to the lack of or unequal distribution of capital. Capital includes financial, human, physical, social, and natural resources. The underlying causes focus on household food security, adequate care and feeding practices, access to health services, and residing in a healthy environment. The immediate causes are the impact of the basic and underlying causes at the individual level through inadequate food intake and disease. Ultimately, the framework provides an interface between these broader systemic-level issues and the community, household, and individual levels. The conceptual framework, originally designed in 1990, has been refined to be broad enough to encompass multiple sectors that impact the causes of malnutrition at each level. The conceptual framework has been modified for specific geographical contexts or with a focus on interventions (5, 12).

Box 1 **Key messages.**

- Chronic undernutrition affects a large number of children under 5 years, especially in developing countries.
- There are many causes of chronic undernutrition, which stem from multiple sectors and often overlap.
- The sectors, which impact chronic undernutrition include agriculture, health, environment, water and sanitation, infrastructure, gender, and education.
- The consequences of undernutrition are both short- and long-term, often having an impact throughout the lifecycle and on future generations.
- There are still many gaps in our knowledge of the causes of undernutrition and the impact of interventions, particularly in the agriculture and environment sectors.

### Basic causes

Historically, policies and programs focused on economic growth have impacted health outcomes through increased access to health services and improved quality of health services. More generally, economic growth, if equitable, leads to greater household purchasing power. Over time, stunting prevalence from 1985 to 2011 across 141 countries showed an overall decline in stunting as economic indicators improved (13). However, an overall analysis of GDP per capita found no significant relationship with reductions in stunting or other nutrition outcomes (14). Again, the increases in wealth at a country level need to be equitable and investments in health and nutrition are needed in order to impact the nutrition status of the most vulnerable households. Furthermore, the level of wealth at the household level has been shown as an indicator for increased risk of stunting (15). There is also an association between increased risk of stunting and lack of basic infrastructure, which includes water, sanitation, electricity, and shelter (16). A similar association between decreased risk of stunting and increased household wealth and improved community resources, specifically health centers, has also been presented in the Young Life Study (17).

Beyond economic and political factors, cultural factors can play an important role in child growth. The cultural factors, which have been shown to be important to the prevalence of stunting, are women’s autonomy and fertility practices. In Indian households, a woman’s status is related to the birth order of either herself or her husband, which can determine the quantity and quality of resources devoted to both the mother and child (18). Additionally, the distribution of resource among children within the same family can differ. In a secondary analysis on prevalence of stunting and birth order, the data showed that sub-Saharan African countries as a whole had a more constant rate of stunting across birth orders compared to India, where higher birth order (i.e., having more older siblings) was associated with an increasing risk of stunting (19). In addition to the birth order of both parents and children, gender can also have a significant influence on growth. In Pakistan, due to the cultural context of feeding and care practice, female children are often considered secondary to male children and were three times as likely to be stunted (20). While cultural factors play an important role in children’s growth and development, the impact of cultural factors on stunting should be considered as being confined to specific geographic areas and ethnic groups rather than as uniform assumptions.

There are certain types of shocks that can cause disruptions in resources and livelihoods, leading to static or worsening prevalence of stunting. Such shocks, including droughts or civil conflicts, lead to increases in stunting in children, particularly in children 12–24 months and in children from poorer households (21). Different types of shocks can impact different cohorts within a population through different mechanisms. A study of conflict and crop failure in Rwanda found that the impact of these events can lead to sustained growth failure, even after the event had ended (22). Climate events and changes to the natural system can affect the prevalence of stunting through both increases in human morbidity and degradation to the local food system and resources. Exposure to droughts and floods in India was shown to have increased the risk of stunting by 7% (23). Exposure to these types of events can be associated with malnutrition through the impact on the quality and quantity of food or can indirectly be associated through the impact on the economy, political systems, public infrastructure, and water systems.

### Underlying causes

The underlying causes of malnutrition, found at the community level, impact the ability of the household and individual to obtain proper nutrition. One underlying cause of malnutrition is inadequate household food security. Food security is characterized by having the combination of available food, access to nutritious foods, and proper utilization of food and can be caused by shocks at the household or community levels either in singular, cyclical, or continuous intervals. At the household level, food insecurity relates more to the household economy and contextual determinants of how food is used and consumed. These determinants include maternal knowledge of care and feeding practices, maternal chores or livelihoods, and family eating behaviors (e.g., who eats first, the size of individual rations, etc.) (24). A study on household food security effects on pre-school children showed that household food insecurity has an effect on the prevalence of diarrhea, which leads to a dose–response effect of diarrhea on the prevalence of stunting (25). At the community level in Bangladesh, food insecurity caused by the monsoon season was associated with wasting, but not with stunting, through decreased dietary diversity and lower household income (26). Furthermore, seasonality has not been shown to affect prevalence of stunting unless seasonality directly affects the disease environment (15). On the other hand, increased prevalence of stunting has been linked to repeat instances of food insecurity through the accumulation of bouts of wasting leading to linear growth failure (27). The biological mechanism behind this is based on the manifestation of wasting causing the body to consume fat and tissue leading to the point when no more fat and tissue can be consumed and there is an interruption of linear growth (28). There are key components that make up a sustainable food system and are closely linked to food security. These components include household income, market prices, gender dynamics, dietary diversity, and household and individual behavior (29). Furthermore, the agricultural systems and environmental components of agriculture systems, such as soil, water, and agrobiodiversity, have an impact on food security through both the quantity of available food and the quality of available food, being both diverse and culturally acceptable (30).

Other underlying causes of malnutrition are inadequate care practices, which include lack of exclusive breastfeeding and poor IYCF practices. Breast milk is important component of an infant’s intake and is key to help build a strong immune system (31). Exclusive breastfeeding for the first 6 months is the UNICEF recommendation (32). Breast milk contains immune-modulating compounds, which are the building blocks to a strong immune system (33). IYCF practices recommend early-initiated and exclusive breastfeeding for the first 6 months, and continued breast-feeding with introduction of adequate solid, semi-solid, and soft food from 6 to 12 months. In a study in Bangladesh of an Integrated Management of Childhood Illnesses (IMCI) strategy, where the IMCI treatment group had a higher prevalence of exclusive breastfeeding for 6 months, exclusive breastfeeding resulted in a 7.3% decrease in the prevalence of stunting (34).

Additional underlying causes of malnutrition are inadequate services and the presence of an unhealthy environment. More specifically, this includes poor access to and quality of health services, water, and sanitation facilities, substandard hygiene practices, and inadequate food preparation, all of which are important in their contribution to the disease environment. A study found that family housing quality was negatively associated with stunting, where housing quality was measured by the type of dwelling, the availability of a safe water supply, the adequacy of sanitation, and garbage collection (35). Poor household environmental conditions were associated with increased infections in children and an increased risk of stunting compared to non-contaminated household environments. (36) There appears to be a positive correlation between water and sanitation hygiene (WASH) and stunting. However, in a literature review of WASH interventions on nutrition status, WASH was only found to be a slightly significant factor in reducing the risk of stunting and was found to increase the average height-for-age *z*-score by 0.08 standard deviations. (37) The authors noted that more rigorous testing was needed. In an additional study, improved sanitation and a reduction in open defecation by 20% was found to increase the average height-for-age *z*-score by 0.1 standard deviations (38).

These underlying causes do not affect stunting in isolation of each other. The combination and interaction between them amplify the effect on the overall prevalence of stunting. A study on the Millennium Villages Project attributes a reduction in stunting to the combination and interaction of several factors across multiple sectors (39). However, further studies are needed to assess the impact of multi-sector efforts on reducing stunting.

### Immediate causes

The immediate causes of the conceptual framework include inadequate food intake and risks posed by the disease environment on the individual. Inadequate food intake refers to both quantity of food and quality of the diet. The nutritional quality of food intake holds importance in driving the biological processes that govern the growth and development of the musculoskeletal and the nervous system. The second part of inadequate food intake is the quality of the food. The quality of a diet is reflected by the dietary diversity and the micronutrient content of the diet. A study in Cambodia showed an association between greater diet diversity and reduction in or lack of stunting (40). A noted key component in this study was the consumption of animal-source foods. Animal-source foods contain vitamin A, vitamin B_12_, riboflavin, calcium, iron, and zinc as well as higher amounts of protein, energy, and fat compared with plant food sources.

The lack of essential vitamins and minerals within the diet is also known as the “hidden hunger.” Deficiency of certain vitamins and nutrients can lead to specific conditions but the deficiency in even just one can be a limiting factor for growth. It is also important to acknowledge that when there is one micronutrient deficiency there is a high likelihood that there are multiple micronutrient deficiencies (7). Research is needed to look at the effect of micronutrient deficiencies on stunting. Furthermore, there are many challenges in diagnosing specific micronutrient deficiencies in low-resource settings, since laboratory testing is often required.

The second immediate cause in the conceptual framework is disease. Disease can be a cause and consequence of malnutrition. Common childhood infections and diarrheal diseases can lead to poor absorption or ability to retain nutrients. The risk of a child being stunted at 2 years of age increases with the incidence of diarrhea (41). The incidence of diarrhea in children has a dose–response effect on stunting; as the incidence of diarrhea increases, the risk of becoming stunted also increases (25). The dose–response effect is part of a downward spiral interaction between poor nutrition and disease.

Children may not only be at risk of stunting due to diarrheal disease but also due to environmental enteropathy. Humphrey proposes environmental enteropathy as a hypothesis, defining environmental enteropathy as a subclinical disease caused by ingestion of fecal material and the subsequent intestinal inflammation and enteric infection (42). There have been few studies examining environmental enteropathy and the link with stunting. Tests for environmental enteropathy include collecting intestinal biopsies and biomarkers to examine the nutrient absorptive capacity of the intestine due to morphological changes in the lining that may take place due to chronic enteric infection (43). A study of Gambian children found no significant association of linear growth with adequate diet or diarrhea but did find an association between poor intestinal absorption and growth faltering (44). There have been few other studies that support the hypothesis of the impact of environmental enteropathy on stunting (45, 46).

Both diarrhea and environmental enteropathy are related to the water and sanitation facilities and practices at the community level. It is important to reiterate that chronic undernutrition through deficiencies of certain nutrients, for example vitamin A and zinc, compromise the immune system, leaving a child more susceptible to infectious diseases including diarrhea, malaria, and measles among other communicable diseases. Pathogens in the environment affect children and can lead to children becoming stunted. In an ecological analysis, a 10% increase in occurrence of open defecation was found to increase stunting by 0.7% points (47). There is a downward spiral of disease and malnutrition that is important to acknowledge as both cause and consequence of chronic malnutrition.

Another contributor to stunting related to quality of food and the disease environment are toxins. This includes both naturally occurring toxins, such as Aflatoxin, and man-made toxins, such as pesticides. For a large portion of toxins, the long-term health impacts on humans are unknown, particularly the impacts on children.

Aflatoxin, a secondary metabolite of *Aspergillus flavus* and *Aspergillus Parasiticus* fungi, is most often found in food crops in tropical and subtropical regions. The most common food crops include maize, groundnuts, oilseeds, and tree nuts. Aflatoxin is produced most frequently during the wet season. Aflatoxin toxicity can be fatal to humans if consumed in high doses. Non-fatal exposure has also been associated with growth impairment in children through immune system suppression, impairment of protein synthesis, and changes in the hepatic metabolism of micronutrients. Exposure to Aflatoxin in children has been identified to occur through passage from the mother, either *in utero* or from breast milk, and through the child’s consumption of complementary foods containing maize and groundnuts (48). More research is needed on the exact mechanisms related to growth impairment from Aflatoxins.

The exposure of both pregnant women and young children to pesticides can have an impact on the growth and development of children. There has been anthropological research showing neurological deficits in children exposed to pesticides (49). This finding points to the need for further clinical research to understand the biological impacts and pathways that pesticides have on children, both in terms of neurological development and the impact on child nutrition. A study on Ecuadorian children discusses the potential compounding effect of pesticides and chronic malnutrition on neurological development (50). The interaction between exposure to toxins and malnutrition on child development is an area in need of further research.

### Timing

Evidence shows that growth faltering can begin *in utero* and continue until approximately 24 months (51). This knowledge has led to what is commonly referred to as the window of opportunity or the first 1,000 days of growth and development. This time period is when a significant amount of critical growth and development takes place. The timing of stunting is not necessarily dictated by the causes but rather by which cause happens at specific growth stages during childhood. Intrauterine growth restriction is a form of chronic undernutrition, which leads to infants being born small or stunted at birth. Intrauterine growth restriction is caused when the mother has not met the nutritional requirements of pregnancy. Maternal body composition (stature and BMI) and mother’s age at childbirth are predictors for stunting (15). In addition, growth failure prior to 12 months of age is most closely associated with low stature of the mother (52).

There is a difference between becoming stunted and already being stunted. In addition to the criteria of falling at or below 2 SD on the height-for-age growth standard, the distinction is particularly important to consider in regards to ability to catch up height later in life. In a study by Mendez and Adair, it was found that children who became stunted earlier *in utero* or in early infancy were more likely to be severely stunted (−3 SD below the mean) (53). Furthermore, the earlier a child becomes stunted, the greater the long-term consequences (9). The timing of becoming stunted earlier in life creates greater potential for detrimental short- and long-term consequences. Stunting beyond the age of 2 years has been shown in children already stunted, suggesting that children who have become stunted in the first 2 years may be chronically at a disadvantage to regain height later in childhood (54).

## Consequences

### Short-term

The consequences of stunting can be both short- and long-term. In the short-term, stunting can result in increased risk of mortality. Mortality is complex to measure, since a child may not die from being stunted as a primary cause. Rather, stunting can be an underlying factor contributing to a child’s vulnerability and susceptibility to morbidities. In 2010, child and maternal undernutrition (including both acute and chronic malnutrition) were responsible for 1.4 million deaths or 6.7% of all DALYs (disability adjusted life years) according to the Global Burden of Disease Study (55). Within the 1.4 million, 0.9 million deaths were due to stunting of children under 5 years. A study of multiple cohorts shows a compounding effect of multiple anthropometric failures (height-for-age, weight-for-age, and weight-for-height) on selective mortality (56).

Morbidity or burden of disease can be caused by both the suppression of the immune system and subsequent long-term chronic health problems. Vitamin A, zinc, and iron are all related to compromised immune function, increased occurrence of infections, and increased risk of stunting (57, 58). Stunting and lowered immune function can lead to higher risk of infectious diseases. This includes susceptibility to diarrhea, as an immediate cause of chronic undernutrition, and other infectious diseases, and can lead to a cyclic effect of infection and malnutrition.

### Long-term

Long-term effects of stunting impact the health, education, and productivity of children as they become adults. One long-term consequence of chronic undernutrition is a decrease in cognitive ability. There has been much research on this association both with educational attainment and wages gained in adulthood. In a study of Peruvian children, chronic malnutrition, as measured by stunting in infancy, leads to poor cognitive development later in childhood (59). Additionally, the Young Lives Study showed that grade achievement and test scores were affected by stunting, but children who were able to catch up in height between 1 and 8 years were also able to catch up cognitively (60). There are several studies that demonstrate the importance of the timing of catch up associated with educational attainment (61, 62). As discussed earlier, interventions in earlier stages of growth and development had larger impacts on long-term wellbeing.

Chronic malnutrition in early childhood not only affects educational attainment but also cognitive ability as an adult and therefore economic earnings in the long-term (63). In rural Zimbabwe, the impact of stunting in pre-school aged children is correlated with lower human capital potential in adulthood (64). Additionally, there has been shown to be an association between linear growth failure at 2 years of age and work status (either working, unemployed, or informal work), whereas stunting at 2 years of age was more closely associated with unemployment or informal work (65). One challenge in measuring employment opportunities and economic capital later in life is due to gender imbalance. While many people work in the informal sector in developing countries, women account for most of this sector, and in some cultural contexts, girls are more likely to suffer from stunting than boys.

Another long-term effect of stunting on morbidity is an increase in the risk of non-communicable diseases such as cardiovascular disease and diabetes related to being overweight or obese. In biological terms, stunting leads to impaired fat oxidation and glucose intolerance (58). Several studies have drawn the association between stunting in early childhood and obesity later in life (66, 67). In some populations, young children have been found to be both stunted and overweight. This relationship, found in rural Mexico, was closely associated with younger mothers, lower socioeconomic status, lower education level of mothers, maternal stature, and household size (68). Furthermore, rapid weight gain and linear growth in children in low and middle-income countries is associated with increased survival and improved cognitive development but may also lead to increased risk of obesity and related adult cardio-metabolic diseases (69). The “thrifty gene” or Barker hypothesis explains that catch up growth later in childhood after experiencing growth failure can increase the risk of adult obesity, which can lead to type-2 diabetes and cardiovascular disease (70). Additionally, research has shown that stunting in early childhood may increase the risk of elevated systolic blood pressure in later life (71). It is important to note that the concept of catch up from being stunted can create some protection from adult chronic disease risk factors. However, the “catch up dilemma” is that increased linear growth can lead to improved health outcomes but too rapid linear growth may be associated with an increased risk for the chronic diseases mentioned (69). This increase in obesity and potential for non-communicable diseases is most often seen in countries facing an epidemiological transition due to globalization, economic growth, and drastic changes in the food environment. Globally, there has been a 54% increase in overweight children under 5 years between 1990 and 2011 (5).

Beyond the long-term effects of stunting, there are effects that transcend generations. Stunted children become stunted adolescents, who then become stunted adults who give birth to stunted infants. More specifically, as mentioned, intrauterine growth restriction can cause the child *in utero* to be small-for-gestational age, which manifests as a low-birth-weight infant at birth. This is most easily seen in the nutritional relationship between mother and child throughout both pregnancy and infancy. If a mother enters her pregnancy with poor nutrition status, she is more likely to give birth to a low-birth-weight or stunted infant (5). Beyond the mother-to-child transmission of nutrition status, there is a cyclical and transgenerational link between agricultural productivity and nutrition status that is important to note (72). The long-term impacts of stunting have a strong impact on agricultural productivity in adulthood, which can further create deficits for future generations. This cycle can be thought of as a nutritional poverty trap. Analysis has shown that the transgenerational effect can be seen through trends in adult height, which can be predicted by height at 2 years (73). Similarly, there have been other papers on the transgenerational consequences of stunting (74, 75).

### Catch up

As discussed, the consequences of stunting can be both detrimental to the individual and community and can have both short-term and long-term impacts throughout the lifecycle. The definition of catch up is a reduction of the stunted child’s height deficit, which occurs in early childhood and involves an increased velocity of linear growth (76). An important question is whether or not there is an opportunity for children to “catch up” in height. An analysis of children from low- and middle-income countries found that 70% of the growth deficit found at the age of 5 years can be attributed to stunting occurring in the first 1,000 days of life (54). Prentice et al. contradict the common belief that there is only one critical window for growth between the ages of conception and 24 months, stating that there are two rapid periods of growth for children, one in early childhood and one during puberty (77). The core argument is that there is a maturational delay in stunted children, which allows growth catch up potential in later childhood by prolonging the pubertal growth phase. Furthermore, there have been documented delays in menarche, which can lead to this opportunity for catch-up growth, particularly in adolescent girls (78).

Timing is critical in terms of achieving catch-up growth. By the age of 3 years, the nutrient requirements drop, making it easier to meet a child’s dietary requirements and making the child less susceptible to chronic undernutrition (9). A study by Outes and Porter in Ethiopia shows that catch up is possible, especially for children from wealthier households, but the window for catch up ended by the age of 5 years (79). Similarly, a study demonstrated that children who displayed catch-up growth had similar cognitive ability later in childhood when compared to children who had never been stunted (80). There are also studies, which have shown that early growth improvements prior to 2 years can have a positive association with school outcomes and cognitive ability (60). Further research is needed to determine the timing and ability of stunted children to catch up both physically and mentally to their non-stunted peers.

## Interventions

The causes and consequences of stunting are both complex and varied. There is no single intervention that will act as a “silver bullet” to solve chronic undernutrition, but rather a multiplicity of interventions as there are multiplicity of causes. There are also many unknowns as to how, when, and which interventions can be most effective. However, there are well-established, key nutrition-specific and -sensitive interventions, including inputs throughout the life cycle and across sectors. There is a need for global goals that have a direct translation to national goals (81). To tackle the challenge of chronic undernutrition, there needs to be assessment of causes, interventions, and advocacy addressing nutrition within the maternal and child health Millennium Development Goals at the national level (82). Both maternal and adolescent girls’ health and nutrition play a seminal role in the nutrition outcomes of children, especially when considering the transgenerational potential of chronic undernutrition. Furthermore, more assessment of interventions through the agriculture and environment sectors is needed to fully address the challenges of malnutrition from a multi-sector perspective.

Most interventions focus on early childhood, specifically on optimal infant and young child nutrition. This time period is not only within the critical window of development but also is an age range that is accessible when integrated with child health days, specifically for measles immunization (83). After birth, IYCF programs and promotion of optimal care practices are important interventions needed to provide adequate diet and a healthy environment for infants and children. The impacts of exclusive breastfeeding, complementary feeding, food supplementation, micronutrient supplementation, family and community health and nutrition practices, and reduction of disease burden on both nutrition status and survival are well documented as to their relationship with reduced stunting (84).

Interventions to improve nutrition practices have been found to be effective. For example, diet diversity for children older than 6 months, measured as consumption of at least four food groups, has been found to have the strongest impact on the prevention of stunting compared to other IYCF interventions (85). As another example, studies have shown that multiple micronutrient supplementation is associated with improvements in height with an effect of 0.13 increase in HAZ between treatment and control groups (86). As yet another example, IMCI interventions that increase exclusive breastfeeding for the first 6 months are associated with a 7.3% decrease in the prevalence of stunting (34). Along with interventions focusing on early childhood health, care, and feeding practices, the addition of mother nutritional education or counseling has a significant impact on reducing stunting, with an added effect size of 0.21 increase in HAZ (87). Furthermore, the promotion of breastfeeding, complementary feeding, and micronutrient supplementation, if scaled to 90% coverage, could lead to a 20.3% decrease in stunted children (88).

To ensure improvements in nutrition, multi-sectoral approaches are vital (89). At minimum, there are three key sectors that need to engage, collaborate, and contribute to nutrition improvements: agriculture, health, and environment sectors that inject nutrition into functioning and effective food, health, and water and sanitation systems (90). The health system has been well defined through nutrition-specific interventions, many of which have been mentioned above. Interventions within the agriculture and environment sectors are less well-known. The heightened global awareness of nutrition points to the need for development institutions and governments to better understand the linkages between multiple sectors and nutrition.

There needs to be greater focus on the linkage between agriculture and nutrition and the ways in which agriculture can contribute to reducing stunting. The “what” and the “how” to effectively deliver “nutrition-sensitive agriculture” services to rural households remains poorly understood. There is a call for “a new emphasis” on nutrition-sensitive agriculture through food and agriculture policies in order to provide scale, coverage, and benefits within nutrition (91). Nutrition-sensitive agriculture involves the design and implementation of nutrition-based approaches to sustainable farming and cropping systems. Ultimately, nutrition-sensitive agriculture is aimed at improving the nutritional status of a population by maximizing the impact of food and of agricultural systems, while minimizing the potential for negative externalities regarding the sector’s economic and production-driven goals. It is agriculture with a nutrition lens, and should not detract from the sector’s own goals (92).

Two recent reviews have examined the impact of agricultural interventions on nutrition outcomes. In one review, agriculture strategies improved dietary patterns and specific micronutrient intakes (vitamin A in particular) in some studies, but there was no significant reductions overall in child growth indicators such as stunting, wasting, and underweight. Some studies individually found reductions in stunting, including a dairy goat project in Ethiopia and a legume intercropping project in Malawi (93). The second review found that agriculture interventions were beneficial in promoting consumption of nutritious foods, but evidence on micronutrient status and growth indicators was not clear or evidence was lacking. This review did show that home garden programs increased the consumption of fruit and vegetables; aquaculture interventions increased the consumption of fish; and dairy projects increased the consumption of milk, improving dietary diversity (94). Furthermore, the addition of milk and other animal-source foods in diets with poor diet diversity decreases the risk of stunting due to the micronutrient content and high-quality protein density of these foods (95). The consumption of wildlife, defined as animal-source food available in the local ecosystem, has been seen to decrease the risk of anemia in children and further protect children from cognitive, motor, and physical deficits (96). Research has also demonstrated a strong association between dietary diversity and diet quality, and nutritional status of children (97–99). It is also clear that household dietary diversity is a sound predictor of the micronutrient density of the diet, particularly for young children (100). In an ideal setting, agriculture can make contributions to dietary diversity by increasing and/or improving diversity of landscapes and the availability of foods produced from those lands. Dietary diversity is a vital element of diet quality. The consumption of a variety of foods across and within food groups, and across different varieties of specific foods, guarantees the adequate intake of essential nutrients and important non-nutrient factors. One recent study demonstrated a positive correlation between crop diversity and dietary diversity (101). More evidence is needed to fully link the impact of biodiversity on nutrition outcomes, which should include better collaboration between health and agriculture (102).

Addressing the nutrition-disease interaction includes interventions such as promotion of handwashing, improved sanitation and water quality, promotion of exclusive breast feeding for the first 6 months, promotion of appropriate complementary feeding including hygienic food handling, increased dietary diversity, and prevention of other diseases such as respiratory tract infections and malaria. As there is clear evidence that the causes of malnutrition have a direct correlation with the WASH environment, it is critical to address. In one study, it was found that hand washing before the preparation of children’s food decreased the incidence of diarrhea. It has been shown that diarrheal incidence increases around 6 months of age when complementary foods are introduced, which is also during the critical period of growth. If WASH interventions can reduce diarrhea, it could have an impact on a child’s cognitive ability, either through an increased ability to attend school or through a decrease in the risk of stunting. Nutrition-specific interventions, if scaled and utilized can have a significant impact on the reduction of stunting, micronutrient deficiencies, and wasting. These interventions target pregnant and lactating women and children under 2 years of age (5, 103, 104). Nutrition-sensitive approaches, while a growing area of focus, address the underlying determinants of malnutrition. Although some sectors still lack evidence, there are some approaches emerging that have enough evidence to scale up in specific-country and local contexts. These include approaches in agriculture, social transfers, early childhood development, and education (91). Although these approaches may not have a direct impact on child stunting or other anthropometric measures, their experimental design is more complex and nuanced as they address the underlying causes of nutrition through a non-linear path that depends strongly on the local context. Many of the nutrition-sensitive interventions fit within a multi-sectoral approach, thus little has been done to unpack how effectively each intervention contributes to a larger multi-sectoral approach, how these different interventions interact with each other, and how they should be more systematically measured when considered as a “package.”

National policy and advocacy are needed to create enabling environments for nutrition action. An analysis of advocacy as intervention shows how cultural and political context play a role in the effectiveness of advocacy (105). Advocacy plays a role in cohesion and coordination between all stakeholders, including governments, international organizations, and donors. Part of strong and effective advocacy is good data collection. Due to the multifaceted nature of chronic malnutrition, nutrition data need to be reflective of indications from all sectors involved, for example, population-level anthropometric data, nutrition-specific data, such as IYCF indicators, and nutrition-sensitive data such as dietary and WASH indicators. Without such data, advocates cannot establish a program’s efficacy.

## Conclusion

This paper reviews the academic literature on current definitions, causes, consequences, and interventions of chronic malnutrition. Additional research is still needed, especially related to the times at which children become stunted and the timing of linear growth catch up. Evidence is also needed on how to increase multi-sectoral nutrition-sensitive interventions (91). This is especially important in understanding the impacts of agriculture and the environment on nutrition, particularly within the context of a changing environment. Most importantly, knowledge is needed on how to effectively scale interventions. A major constraint of current interventions is related to capacity and human resources (106). Strengthening capacity and scaling interventions will be core components of all national nutrition systems and will involve integration with all sectors, including health, agriculture, environment, finance, trade, public infrastructure, education, early childhood development, and gender, to name a few. Political will, commitment, and collaboration are required from governments, as well as increased commitment and collaboration from the international and donor communities.

Addressing the burden of stunting is inextricably linked to wider progress toward the Millennium Development Goal targets ending in 2015, the new Sustainable Development Goals post-2015, and overall development of nations. Significant gains will hinge on concurrent steps to reduce poverty, improve access to education, empower women and girls, and facilitate access to basic infrastructure including safe water and sanitation, energy, transport, and communication. Working on multiple fronts simultaneously has the potential to leverage synergies and catalyze gains that extend beyond those achieved through sector specific programs working in isolation (107).

## Conflict of Interest Statement

The authors declare that the research was conducted in the absence of any commercial or financial relationships that could be construed as a potential conflict of interest.
